# Reply to Schulte-Pelkum, J. Comment on “Favresse et al. Persistence of Anti-SARS-CoV-2 Antibodies Depends on the Analytical Kit: A Report for Up to 10 Months after Infection. *Microorganisms* 2021, *9*, 556”

**DOI:** 10.3390/microorganisms9091849

**Published:** 2021-08-31

**Authors:** Julien Favresse, Constant Gillot, Jonathan Douxfils

**Affiliations:** 1 Department of Laboratory Medicine, Clinique St-Luc Bouge, 5004 Namur, Belgium; 2 Department of Pharmacy, Namur Research Institute for Life Sciences, University of Namur, 5000 Namur, Belgium; constant.gillot@unamur.be (C.G.); jonathan.douxfils@unamur.be (J.D.); 3 Qualiblood s.a., 5000 Namur, Belgium

## Reply to the Response Letter

We thank the authors of this Response Letter for their comment on our previous article [1]. In our cohort of 188 samples collected >15 days since diagnosis in patients with a documented positive SARS-CoV-2 RT-PCR, the EliA SARS-CoV-2-Sp1 IgG test (or Phadia S1 IgG) showed a sensitivity of 69.7%, leading to 57 false negative samples. The false negative samples were reported since day 16 to day 297. This sensitivity was lower compared to the five other immunoassays tested in our study [1]. The fact that more than 30% of the samples with a confirmed RT-PCR were not detected as positive led us to propose a cut-off optimization, as successfully performed previously for other anti-SARS-CoV-2 assays [2,3].

Based on our suggestion, the manufacturer proposed a new cut-off of 0.7 U/mL (Response Letter). We thus recalculated the sensitivity using exactly the same cohort and we obtained a sensitivity of 97.3% (95% CI: 93.9%–99.1%; 5 false negative results), which is in line with other immunoassays [1] and with the sensitivity reported by the manufacturer, i.e., 99.4% (95% CI: 98.5%–99.8%) (Response Letter). According to the manufacturer, the specificity slightly decreased from 99.4% (95% CI: 97.9%–99.8%) to 97.9% (95% CI: 96.2%–98.9%).

As proposed by the authors of this Response Letter, we also measured the neutralizing antibodies using a surrogate virus neutralization test (sVNT), an ACE2 receptor binding inhibition assay (cut-off of 10 AU/mL; Shenzhen YHLO Biotech Co., Ltd., Shenzhen, China). For that purpose, 150 remaining samples from our previous study were tested. Among them, 144 samples were collected >15 days since diagnosis [1]. The sVNT was also compared to a pseudo-virus neutralization test (pVNT) developed by the University of Namur on a validation cohort composed of 71 samples (23 pre-pandemic samples and 48 samples from COVID-19 patients) [4]. Our preliminary results confirm that the sVNT and the pVNT assays had a very good agreement (97.2%), as observed by Tan et al. [5].

The EliA SARS-CoV-2-Sp1 IgG test has a highly significant correlation with the sVNT assay (r = 0.89; P < 0.0001) and at the IFU cut-off of 10 U/mL, the agreement with the sVNT was 74.7%. With the adapted cut-off of 0.7 U/mL proposed in this Response Letter, the agreement increases to 87.3% with 17 samples being negative with the sVNT but positive with the IgG assay, and 2 samples being positive with the sVNT and negative with the IgG assay.

We agree that measurement of neutralizing antibodies is of importance because they represent the real protective immunity of antibodies [6]. Different cut-offs could be set up depending on the aim of the analytical kit, i.e., assessment of seroprevalence or tentative to evaluate the neutralizing capacity. With the increasing number of subjects being vaccinated, analytical kits that use a spike antigen can no longer discriminate seroprevalence from SARS-CoV-2 infection or vaccination. Therefore, one reliable strategy for the developers of these analytical kits is to identify the threshold that is the most predictive of the neutralizing capacity.

Interestingly, a significant and continuous decrease of neutralizing antibodies was observed over time (r = −0.21; P = 0.012) using the sVNT assay which is in line with the serological assays. Nevertheless, if the residual antibody response drives the strategy and/or the administration for a boost dose of the vaccine, the use of sVNT (or pVNT) assays should be preferred, especially since they are now implementable on several automated platforms. In addition, they could be adapted to assess specifically the neutralizing capacity towards mutated spike proteins observed in variant strains.
